# Author Correction: Comparative analysis of dimension reduction methods for cytometry by time-of-flight data

**DOI:** 10.1038/s41467-024-47234-3

**Published:** 2024-04-08

**Authors:** Kaiwen Wang, Yuqiu Yang, Fangjiang Wu, Bing Song, Xinlei Wang, Tao Wang

**Affiliations:** 1 Department of Statistical Science, Southern Methodist University, Dallas, TX 75275 USA; 2 Quantitative Biomedical Research Center, Peter O’Donnell Jr. School of Public Health, University of Texas Southwestern Medical Center, Dallas, TX 75390 USA; 3 Department of Mathematics, University of Texas at Arlington, Arlington, TX 76019 USA; 4 Center for the Genetics of Host Defense, University of Texas Southwestern Medical Center, Dallas, TX 75390 USA; 5 Center for Data Science Research and Education, College of Science, University of Texas at Arlington, Arlington, 76019 USA

**Keywords:** Software, Standards, Immunology, Cytological techniques, Quality control

Correction to: *Nature Communications* 10.1038/s41467-023-37478-w, published online 01 April 2023

The original version of this Article omitted a reference to previous work in ‘Lambert et al., 2022’. This has been added as reference^20^ in the Introduction, in the text: “With the rise of scRNA-seq, numerous new DR algorithms, such as tSNE^13^, UMAP^14^, SAUCIE^15^, ZIFA^16^, PHATE^17^, scvis^18^, Diffusion map^19^, and SQuaD-MDS^20^ have been developed”. This has been corrected in the PDF and HTML versions of the Article.

